# Spinal Schwannoma Missed on Chest MRI: A Case of Diagnostic Oversight Prevented by Clinical Re-evaluation

**DOI:** 10.7759/cureus.82568

**Published:** 2025-04-19

**Authors:** Kouichi Asahi

**Affiliations:** 1 Internal Medicine and Pediatrics, Kohokuekimae Ohisama Clinic, Tokyo, JPN; 2 General Medicine and Radiology, Dokkyo Medical University Saitama Medical Center, Saitama, JPN

**Keywords:** clinical re-evaluation, diagnostic error, field of view (fov) limitation, mri interpretation, spinal schwannoma

## Abstract

Spinal schwannomas are benign tumors that can cause significant morbidity if undiagnosed. Misdiagnosis may occur when imaging has a narrow field of view (FOV), especially if clinicians overly rely on radiology interpretations without correlating clinical findings. Here, we report a case of a 58-year-old man who presented with chronic chest and back pain initially attributed to musculoskeletal causes. A non-contrast chest MRI from another hospital was reported as normal. Upon presentation, neurological findings revealed sensory deficits over thoracic dermatomes. The attending physician, board-certified in both general internal medicine and diagnostic radiology, identified a subtle lesion at the edge of the original imaging field. A dedicated contrast-enhanced thoracic spine MRI revealed a tumor consistent with spinal schwannoma at the T7 level. Surgical resection resulted in complete symptom resolution. This case highlights how clinical re-evaluation and imaging reassessment can prevent diagnostic errors associated with a limited FOV and cognitive biases.

## Introduction

Diagnostic errors are a persistent challenge in clinical practice, particularly in radiology. Studies estimate that such errors occur in 3%-6% of all radiologic interpretations [1], with field-of-view (FOV) limitations and cognitive biases among the contributing factors [2]. Spinal schwannomas are benign nerve sheath tumors that represent approximately 25%-30% of all intradural extramedullary spinal tumors [3]. They typically present in adults between the ages 40 and 60 years, with symptoms including localized back pain, radiculopathy, and sensory disturbances. Due to their slow growth, early symptoms may be subtle and nonspecific, leading to diagnostic delays. Differential diagnoses include meningiomas, metastases, and neurofibromas. MRI remains the gold standard for diagnosis, and gross total resection is the preferred treatment with good long-term outcomes [4]. Here, we present a case of spinal schwannoma that was missed on a chest MRI scan due to limited imaging scope, but later identified through clinical re-evaluation by a generalist physician with radiology training.

## Case presentation

A 58-year-old man presented to the department of general medicine with a three-year history of persistent left-sided chest and upper back pain, exacerbated by coughing, sneezing, and movement. He had previously been evaluated by an orthopedic specialist and received symptomatic musculoskeletal therapy, but without improvement. A non-contrast chest MRI, performed at an outside facility to assess nonspecific thoracic abnormalities including pleural, rib, or intercostal pathology, was interpreted as unremarkable.

**Figure 1:**
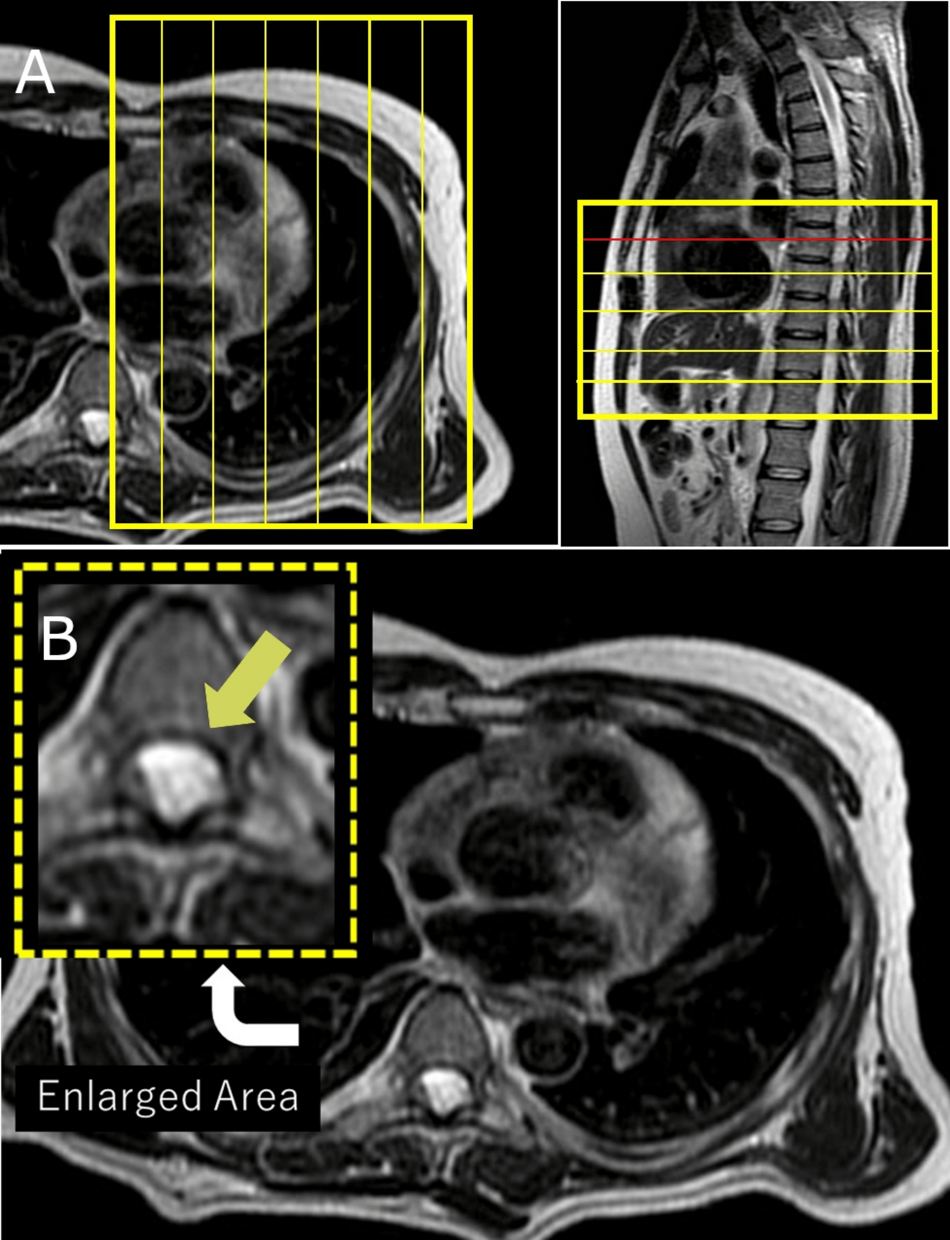
Initial chest MRI with a limited field of view (A) Initial chest MRI (sagittal and axial views) acquired at an external institution: the field of view (FOV) was focused primarily on the thoracic cavity, excluding adequate visualization of the spinal canal. In the sagittal image, the spinal canal and nerve roots fall outside the FOV, and only a single axial slice incidentally captures the lesion at the uppermost level. (B) Axial T2-weighted MRI at the T7 level: a faint hyperintense lesion is seen in the posterior aspect of the spinal canal. The inset highlights the unreported lesion (arrow), which was initially overlooked due to its peripheral location within the limited imaging field.

At the time of presentation, the patient reported symptom progression along with new-onset numbness in both calves. A neurological examination revealed decreased sensation over the T6-T8 dermatomes, with preserved motor strength and normal deep tendon reflexes. The referring MRI was re-reviewed by the attending general internist, who is also board-certified in diagnostic radiology with subspecialty expertise in neuroradiology. A subtle T2 hyperintensity was identified along the posterior margin of the spinal canal near the T7 level, positioned at the periphery of the imaging FOV (Figure 1).

**Figure 2:**
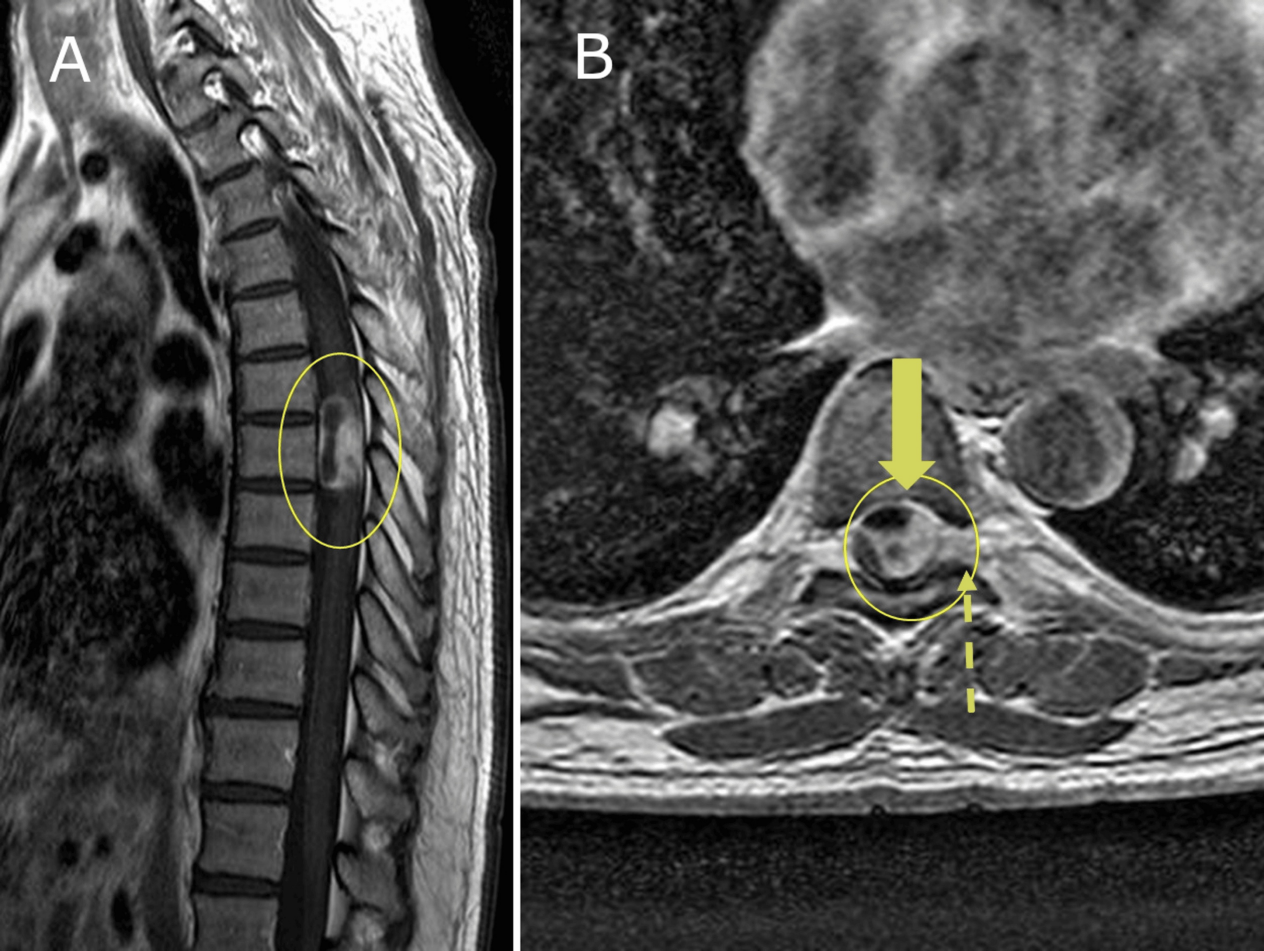
Contrast-enhanced thoracic spine MRI revealing a spinal canal tumor consistent with a schwannoma (A) Sagittal T1-weighted post-gadolinium MRI showing a well-defined, avidly enhancing intradural extramedullary tumor compressing the spinal cord at the T7 level (yellow oval). (B) Axial post-contrast image at the same level reveals strong enhancement of the lesion (bold arrow), with slight lateral extension beyond the dural margin (dashed arrow), a characteristic feature of spinal schwannomas. These findings were consistent with the surgical and histopathological diagnosis.

A contrast-enhanced thoracic spine MRI revealed a well-defined, avidly enhancing tumor within the spinal canal at the T7 level, measuring approximately 1.8 cm. The lesion appeared isointense on T1-weighted images, hyperintense on T2-weighted images, and demonstrated strong post-gadolinium enhancement (Figure 2). These imaging findings were highly suggestive of a spinal schwannoma.

**Figure 3:**
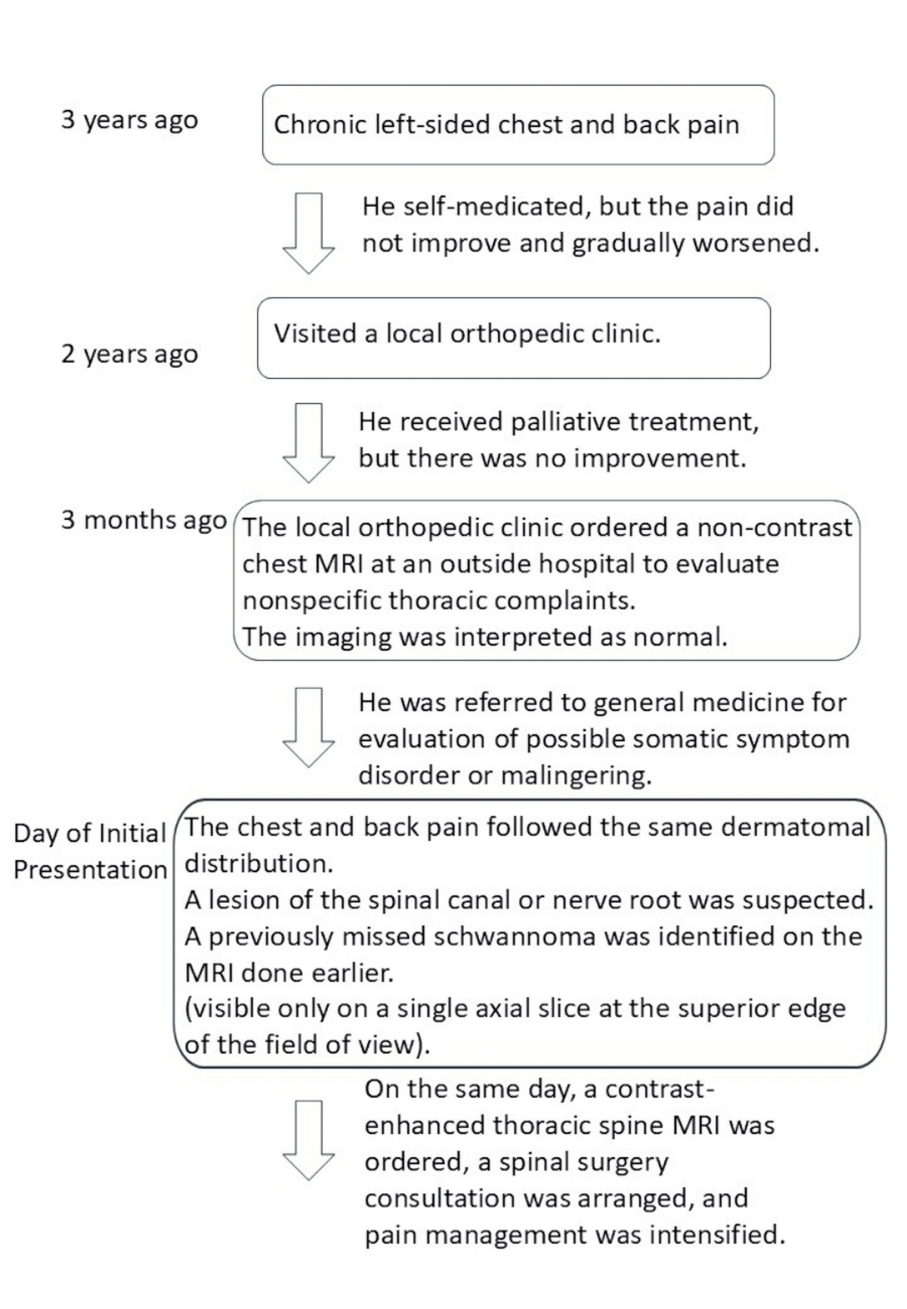
Timeline of the three-year clinical course and diagnostic process Initial symptoms were managed conservatively. A chest MRI ordered two years earlier was read as normal. Upon referral to general medicine, a dermatomal pattern raised suspicion for a spinal lesion. A missed schwannoma was identified on prior imaging. On the same day, contrast-enhanced MRI, surgical consultation, and pain management were initiated.

These imaging findings were highly suggestive of a spinal schwannoma. A timeline of the clinical course and diagnostic steps is shown in Figure 3.

The patient underwent successful surgical resection at a referral institution. The neurosurgical team confirmed the diagnosis of spinal schwannoma based on intraoperative findings and histopathological evaluation. Although the schwannoma was pathologically confirmed, the histological images could not be obtained for publication due to institutional restrictions. The imaging characteristics, including avid contrast enhancement, location within the spinal canal, and slight lateral extension, were highly consistent with a spinal schwannoma. His postoperative recovery was uneventful, with complete resolution of both pain and sensory symptoms.

## Discussion

This case illustrates how diagnostic errors may result from both technical limitations in imaging and cognitive factors. FOV limitations are well-documented contributors to missed diagnoses, particularly when the imaging protocol targets anatomical regions unrelated to the underlying pathology [2,3]. In this instance, the chest MRI was not optimized for spinal evaluation, and the report did not acknowledge this limitation, potentially contributing to false reassurance [1].

Overreliance on radiology reports, especially when they fail to address technical limitations, can lead to overconfidence and premature diagnostic closure, a form of cognitive bias [5,6]. The reassessment of imaging in the context of evolving clinical signs serves as a critical safeguard against such errors. General physicians, with training in holistic assessment and increasing familiarity with imaging interpretation, are uniquely positioned to identify diagnostic mismatches and challenge initial assumptions [7].

Furthermore, this case underscores the importance of correlating neurological findings with imaging results. Even subtle sensory deficits may indicate significant spinal pathology. When discrepancies arise between clinical findings and radiologic interpretations, further investigation should be pursued without delay. Missed or delayed recognition of spinal schwannoma may result in progressive and potentially irreversible neurologic impairment [8,9]. In this case, constructing a clinical timeline clarified the missed opportunities and highlighted key decision points where re-evaluation altered the diagnostic trajectory.

## Conclusions

Spinal schwannomas can be easily overlooked when imaging does not adequately include the spinal canal or when radiology reports are accepted without a critical review. This case underscores the importance of correlating physical examination findings with imaging results and re-evaluating prior studies as clinical symptoms evolve. A heightened awareness of field-of-view limitations and cognitive biases is essential to avoid diagnostic errors and ensure timely and accurate intervention.
